# Association of the *CYP4V2* polymorphism rs13146272 with venous thromboembolism in a Chinese population

**DOI:** 10.1007/s10238-018-0529-y

**Published:** 2018-10-01

**Authors:** Yongjian Yue, Qing Sun, Chiwai Man, Yingyun Fu

**Affiliations:** 1 Institute of Shenzhen Respiratory Diseases, Department of Respiratory and Critical Medicine, The Second Clinical Medical College (Shenzhen People’s Hospital) of Jinan University, No. 1017 Dongmen North Road, Luohu District, Shenzhen, 518020 Guangdong China; 2 Shenzhen Key Laboratory of Reproductive Immunology for Peri-implantation, Fertility Center, Shenzhen Zhongshan Urology Hospital, Shenzhen, Guangdong China; 3 Department of Orthopaedics and Traumatology, Faculty of Medicine, The Prince of Wales Hospital, The Chinese University of Hong Kong, Shatin, Hong Kong, HKSAR, China

**Keywords:** Genetic association studies, *CYP4V2*, rs13146272, Venous thromboembolism

## Abstract

Genome-wide association studies have identified the *CYP4V2* polymorphism (rs13146272) as a risk factor associated with venous thromboembolism (VTE). However, due to the small sample size and variance in genetic analysis models, the relationship between VTE and rs13146272 remains unclear. Here, we performed a case–control study to analyse the associations between rs13146272 and VTE in a Chinese population and to compare the differences among various ethnicities. In this study, 226 VTE patients and 205 healthy controls were recruited, and the allele frequency of variant rs13146272 was analysed by a MassARRAY SNP genotyping assay. In addition, 9 case–control cohorts from 5 studies involving 6667 VTE-affected individuals and 8716 control subjects were included in this meta-analysis. Pooled ORs and 95% CIs were calculated to assess the association between rs13146272 and VTE by using different genetic models. Our case–control study results showed that there was no significant association between VTE and rs13146272 under the additive model (OR = 0.92, 95% CIs: 0.70–1.21, *p* = 0.55) in this Chinese population. However, the results of the meta-analysis performed by merging all cohorts showed that rs13146272 was significantly associated with VTE under the additive model, recessive model and dominant model. In the additive and recessive models, the association reached the threshold for genome-wide significance (*p* < 5.0e^−08^). In conclusion, our pooled systematic study results indicated that individuals with the A allele had a higher risk of developing VTE than those with the C allele of the rs13146272 variant, but the risk was inconsistent among different ethnicities. Further validation of this association with larger sample sizes and multiple ethnicities is warranted.

## Introduction

Venous thromboembolism (VTE) is a complex and multi-factorial thrombotic disorder that includes deep venous thrombosis (DVT) and pulmonary embolism (PTE). VTE is a common cause of morbidity and mortality in adult patients. The incidence of VTE exceeds 1 per 1000 in Caucasians; the incidence among Africans and Asians may be lower and higher, respectively [1, 2]. Among all VTE patients, approximately 30% die within 30 days, and 25% suffer from sudden death [3, 4]. In the USA, approximately 0.6 million new cases of DVT are diagnosed annually [3]. Various risk factors and interactions between acquired and inherited predispositions to thrombosis are suggested to contribute to VTE development [5, 6]. The common risk factors for VTE include multiple surgeries, multiple traumas, fracture, neurological paralysis, cardiac or respiratory failure and various inherited and acquired haematological conditions [6, 7]. Previous studies have shown that ABO blood types are associated with the risk of venous thromboembolism [8, 9]. Studies have also reported that the risk of developing VTE in an individual with an affected sibling was 2.5 times higher than the risk in the general population [10]. Genetic factors, such as hereditary coagulopathies, genetic-associated variants and susceptibility genes, have been linked to the pathogenesis of VTE [11, 12].

Genetic association studies have identified many candidate variants, including *Factor V Leiden*, *FII* (G20210A) and *MTHFR* (C677T) [13–16]. Previous studies showed that rs13146272 was associated with DVT and factor XI levels [17]. The SNP (single nucleotide polymorphism) of rs13146272 is located in the coding region of cytochrome P450 family 4 subfamily V member 2 (*CYP4V2*), which is close to the gene encoding coagulation factor XI. Although *CYP4V2* has been shown to be involved in lipid metabolism, little is known about its involvement in the pathogenesis of VTE [18]. In addition, haplotype analysis indicated that the association between rs13146272 and DVT may be coincident with the risk allele of two SNPs (LD: *r* < 0.12) in the coagulation gene of factor XI [19].

To the best of our knowledge, the association between rs13146272 and VTE has not been confirmed due to the inconsistent findings among different case–control studies [20, 21]. One study showed that the statistically significant association between rs13146272 and VTE was lost after correction for multiple factors [22]. Although previous studies have shown a significant association between rs13146272 and VTE (*p* < 1.7e^−07^), this value is still far from the threshold for genome-wide significance (*p* < 5.0e^−08^) [21, 23]. In addition, a meta-analysis study by Jiang et al. [24] included only partial genotype data for rs13146272, possibly leading to biased outcomes. In addition, most previous studies used only the additive model for genetic analysis to evaluate the odds ratio of the variants in VTE. Therefore, to provide reliable risk estimates and convincing evidence of the association between the *CYP4V2* allele and VTE, we performed a case–control study and meta-analysis to investigate the association of rs13146272 allelic and genotypic frequencies with VTE risk by using different models for genetic analysis.

## Materials and methods

### Study subjects

A total of 226 VTE patients and 205 healthy controls were recruited from December 2013 to September 2017 at the Second Clinical Medical College of Jinan University (Shenzhen People’s Hospital). The PTE patients were diagnosed according to the criteria released by the European Society of Cardiology (ESC) published in 2014 [25]. Three groups of subjects were recruited as healthy controls. The first group included healthy siblings from the PTE family; the second group included healthy subjects according to physical examination with matched ages; and the third group included healthy subjects (age was not matched) without any cardiovascular diseases among three generations of their family. Informed consent was obtained from each patient and each healthy individual before enrolment in the study. This study was approved by the Ethics Committee of Shenzhen People’s Hospital.

### Inherited risk factor assay

Common genetic risk factors were evaluated, and the activity deficiency subjects were considered to have inherited risk factors. Since factor V Leiden and G20210A are very rare in the Chinese population, only the loss of function mutations involving natural anticoagulant factors was evaluated. Blood samples of VTE patients were collected into Na-citrate-containing vacutainer tubes. Plasma was obtained by centrifugation at 4 °C for 15 min at 2000 g and stored at − 80 °C until use. The protein C (*PROC*) and antithrombin (*SERPINC1*) activity levels were determined by the chromogenic method assay on an IL coagulation system (Werfen, Bedford, USA) according to the manufacturers’ instructions. Their activity was quantified with a synthetic chromogenic substrate. Then, the absorbance was detected at 405 nm on a Werfen ACLTOP 700 system (Bedford, USA). The protein S activity was correlated with the prolongation of the clotting time. The normal range of *PROS1* was evaluated using the protein S activity kit on representative members with the ACL TOP family systems. The normal range of *PROC* and antithrombin activity levels were based on hundreds of clinical subjects.

### Genotyping by the Sequenom platform

Genomic DNA was extracted from whole blood using the QIAamp DNA Blood Kit (QIAGEN, Hilden, Germany). The candidate SNP was genotyped by a MassARRAY genotyping assay (Sequenom, San Diego, USA). The PCR amplification primers were designed by Sequenom’s MassARRAY Designer software. The amplification primers for rs13146272 were as follows: forward, 5′-ACGTTGGATGGGCTTGATCTCTGGTACCTT-3′ and reverse, 5′-ACGTTGGATGTCAGGGACTTACACTGTTGG-3′. The genotyping assay extension primer for the SNP rs13146272 was 5′-AGGAACACAAAAAGAGCCTTC-3′.

### Systematic literature search strategy

A comprehensive literature search for suitable studies published up to March 2018 was conducted in PubMed, Science Direct, ISI Web of Knowledge, Google scholar and the Chinese database CNKI. Search terms using the Boolean operator in PubMed included but were not limited to the following: (“venous thrombosis” [MeSH Terms] OR VTE[All Fields]) AND (*CYP4V2* [MeSH Terms] OR rs13146272 [All Fields]). The same keywords were also used for the searches at the other databases. Articles published in English or Chinese with English abstracts were included in the meta-analysis.

### Eligibility criteria

The criteria for including eligible studies were (1) the DVT or PE should be diagnosed with standard criteria in the case group; (2) studies should explore the association between rs13146272 and VTE; (3) variant genotype frequency should be examined with case–control groups. For exclusion, reviews, conference abstracts, republished or duplicate studies and meta-analyses were not considered eligible. We included only the largest data set when more than one study demonstrated the same case–control group of the variant genotype. Animal studies and studies without sufficient variant genotype data were also excluded.

### Data extraction and validity assessment

The following information was extracted from each eligible study: name of the first authors, year of publication, name of study cohorts, ethnicity, genotype methods and distribution of variants. The validity of data was evaluated by two independent reviewers (YY & QS), and discrepancies were resolved by a third reviewer (YF). If the genotype data of the variants were not complete or available in the eligible publications, the first or corresponding author of the studies was contacted to send their datasets via email. The genotype data of publications were excluded if polymorphic variants in the control population did not follow the Hardy–Weinberg equilibrium (HWE).

### Statistical analysis

All statistical analyses were performed using STATA 14.0 (College Station, USA) and SPSS 19 (IBM, New York, USA) software. The OR and 95% CIs were measured to evaluate the strength of the association by a fixed-effects model (Mantel–Haenszel method) or random-effects model. The *z* test was used to evaluate the significance of the pooled OR, and *p* < 0.05 was considered statistically significant. Three genetic models, the additive model, dominant model and recessive model, were used to assess the association between the variant and VTE [26]. Heterogeneity across the included studies was tested by Cochran’s Q statistic and the *I*^2^ statistic, and *p* < 0.05 was considered statistically significant. *I*^2^ < 50% indicated a low heterogeneity level, and a fixed-effects model was used to evaluate the effect. *I*^2^ ≥ 50% indicated high heterogeneity, and a random-effects model was used for the analysis [27]. Egger’s and Galbraith’s tests were employed to assess publication bias [28, 29], and *p* < 0.05 was considered statistically significant. Sensitivity analysis was conducted by omitting one study from the pooled studies at each time point. Differences in age between the VTE and control groups were assessed by Student’s t test. The Chi-square test was performed to compare categorical variables.

## Results

### Characteristics of the recruited participants

The general characteristics of the VTE patients and healthy controls are presented in Table 1. Five DVT subjects and 221 PTE cases were recruited. The five DVT subjects were enrolled as they were siblings of PTE subjects. The age distribution of the patients with VTE was approximately 59.0 ± 1.3 years old (*p* < 0.05). Because some patients refused to participate in all clinical examinations, only the known clinical risk factors, including hypertension, diabetes, cancer and cardiovascular disease, are presented in Table 1. Those subjects who did not carry any acquired thrombophilia risk factors were considered inherited risk VTE cases. A total of 67 inherited VTE patients were recruited. In total, 226 VTE patients were genotyped by the MassARRAY platform. The allele and genotype frequencies of the *CYP4V2* polymorphism (rs13146272) in VTE patients and controls are summarized in Table 1.

**Table 1:** Demographics of the VTE participants, healthy controls and genotype frequency of rs2227589

Groups	Catalogues	Case (*n* = 226)	Control (*n* = 205)	*p* values
Gender	Male/female (*n*)	117/109	97/108	> 0.05
Age	Years	59 ± 1.3	41 ± 1.3	< 0.05
Risk factors	Inherited	67	–	
	*PROC* deficiency	29	–	
	*PROS1* deficiency	28	–	
	*SERPINC1* deficiency	25	–	
	Family history of VTE	13	–	
	Cancer	19	–	
	Cardiovascular disease	41	–	
	Hypertension	83	–	
	Surgery, immobilization	34	–	
	Diabetes mellitus	24	–	
Genotype of rs2227589s	AA	45	38	
	AC	107	111	
	CC	74	56	

### Association analysis of rs13146272 with VTE by a case–control study

**Fig. 1:**
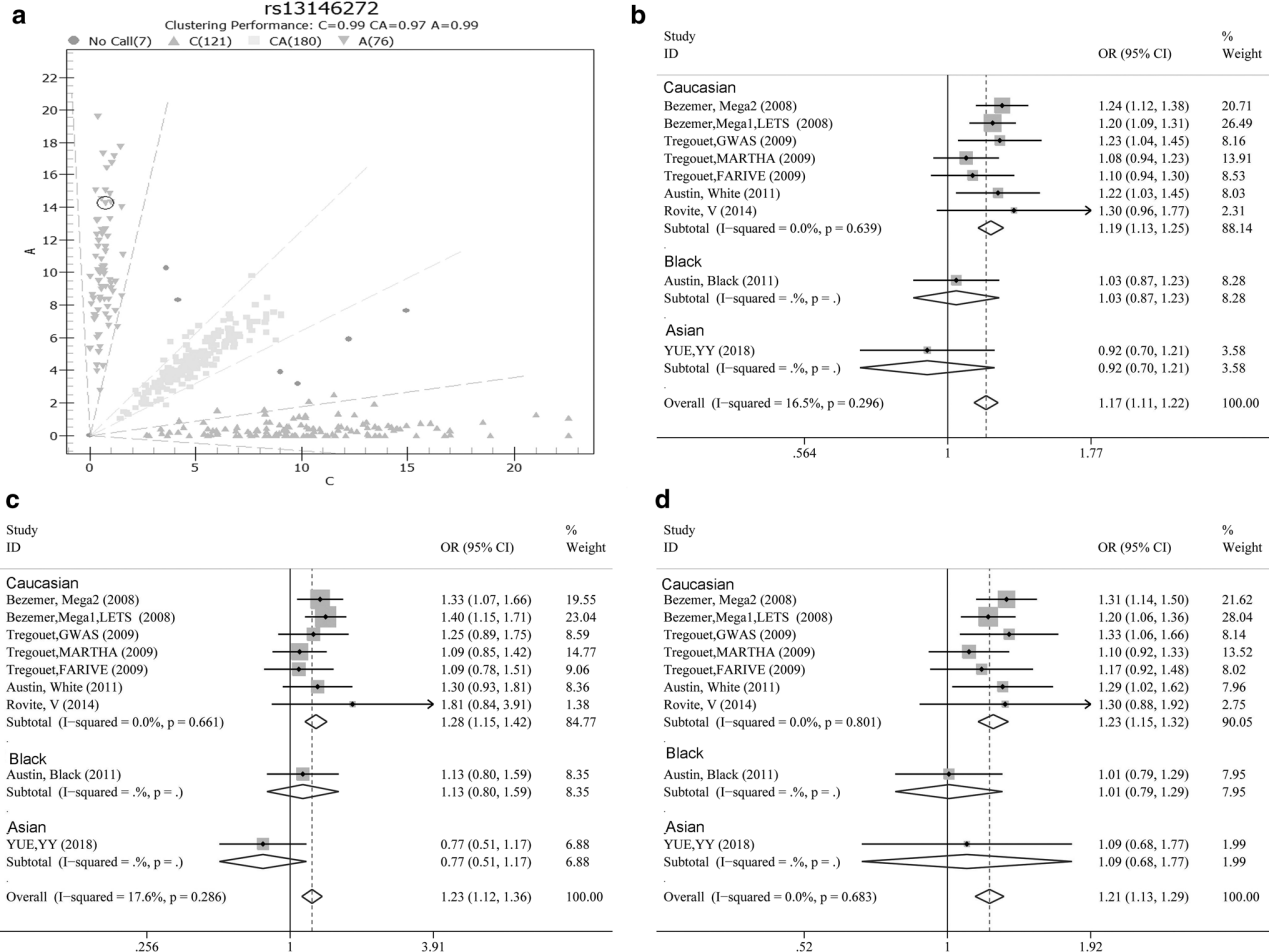
**a** Genotype distribution of rs13146272; forest plots for the meta-analysis of the rs13146272 polymorphism with different genetic models: **b** additive model, **c** dominant genetic model and **d** recessive model

In our case–control study, the CC genotype (minor allele) was found in 48.6% of patients, which was much higher than the value in previous reports (approximately 15%) (Fig. 1). Both the case and control groups showed similar frequencies of SNP homozygotes and heterozygotes. Our case–control study showed no significant association between rs13146272 and VTE under the additive model (OR = 0.92, 95% CIs: 0.70–1.21, *p* = 0.55), recessive model (OR = 1.09, 95% CIs: 0.68–1.77, *p* = 0.72) or dominant model (OR = 0.77, 95% CIs: 0.51–1.17, *p* = 0.22). The models for genetic analysis showed that the *CYP4V2* polymorphism (rs13146272) was not statistically significantly associated with an increased risk of VTE in this Chinese population.

### Characteristics of eligible studies

In the present study, we included 9 case–control cohorts from 5 studies with 6667 VTE-affected individuals and 8716 control subjects for the meta-analysis. The genotype data were kindly shared by the author (Pierre-Emmanuel Morange), and the genotype data were not included in the analyses of previous publications [21]. The summarized information of the eligible studies is shown in Table 2. All the variant genotypes in the control group from the 9 cohorts met the HWE criteria (*p* > 0.05). The genotype distribution and allelic frequencies of all eligible studies are presented in Table 2.

**Table 2:** Allelic frequencies and characteristics of the eligible studies

Authors	Years	Groups	Genotyping methods	Case			Control			HWE
				CC	CA	AA	CC	CA	AA	
Bezemer et al.	2008	Mega2	PCR	121	478	561	352	1178	1094	0.22
Bezemer et al.	2008	Mega1, LETS	PCR	181	808	850	293	995	919	0.36
Tregouet et al.	2009	GWAS	Beadchip	50	170	191	181	561	486	0.36
Tregouet et al.	2009	MARTHA	Tapman	160	547	481	116	377	304	0.96
Tregouet et al.	2009	FARIVE	Tapman	79	277	237	84	290	213	0.35
Austin et al.	2011	White American	Tapman	67	233	244	102	303	256	0.43
Austin et al.	2011	Black American	Tapman	70	264	195	84	278	210	0.61
Rovite et al.	2014	Latvia	Tapman	10	71	96	23	100	112	0.92
Yue et al.	2018	Asian	Sequenom	74	107	45	56	111	38	0.54

### Meta-analysis of the association between rs13146272 and VTE

Meta-analysis was performed in conformity with the statements of Preferred Reporting Items for Systematic Reviews and Meta-Analyses (PRISMA) [30]. We used the major allele for the SNP (A allele) as the risk allele for the meta-analysis. All models for genetic analysis showed *I*^2^ < 25% and *p* > 0.05, which indicates that our studies have very low heterogeneity. Thus, we used a fixed model for further analysis. The association effect distribution is presented in forest plots (Fig. 1). The association effect of the pooled OR was 1.17 with the additive model (95% CIs: 1.11–1.22, *p* = 4.3e^−10^), was 1.23 the dominant model (95% CIs: 1.12–1.36, *p* = 2.6e^−5^) and was 1.21 the recessive model (95% CIs: 1.13–1.29, *p* = 1.1e^−8^). The association effects among rs13146272 and VTE for all genetic models are shown in Table 3. Our pooled systematic results demonstrated that SNP rs13146272 was significantly associated with the risk of developing VTE. There was a significant association between rs13146272 and the risk of VTE under all genetic models (Table 3). Subgroup analysis by ethnicity showed that rs13146272 was significantly associated with the risk of developing VTE in white groups (Fig. 1).

**Table 3:** The results of pooled OR, 95% CIs and heterogeneity by meta-analysis

Genetic models		Pooled effect		*z*	Heterogeneity		Galbraith
Models	Allele	OR (95% CIs)	*P*_*z*_		*I*^2^ (%)	*P*_H_	*p*
Additive	A versus C	1.17 (1.11–1.22)	4.3e−10	6.24	16.5	0.30	0.73
Dominant	AA + AC versus CC	1.23 (1.12–1.36)	2.6e−5	4.20	17.6	0.29	0.37
Recessive	AA versus AC + CC	1.21 (1.13–1.29)	1.1e−8	5.71	0.0	0.68	0.94

### Publication bias and sensitivity analysis

**Fig. 2:**
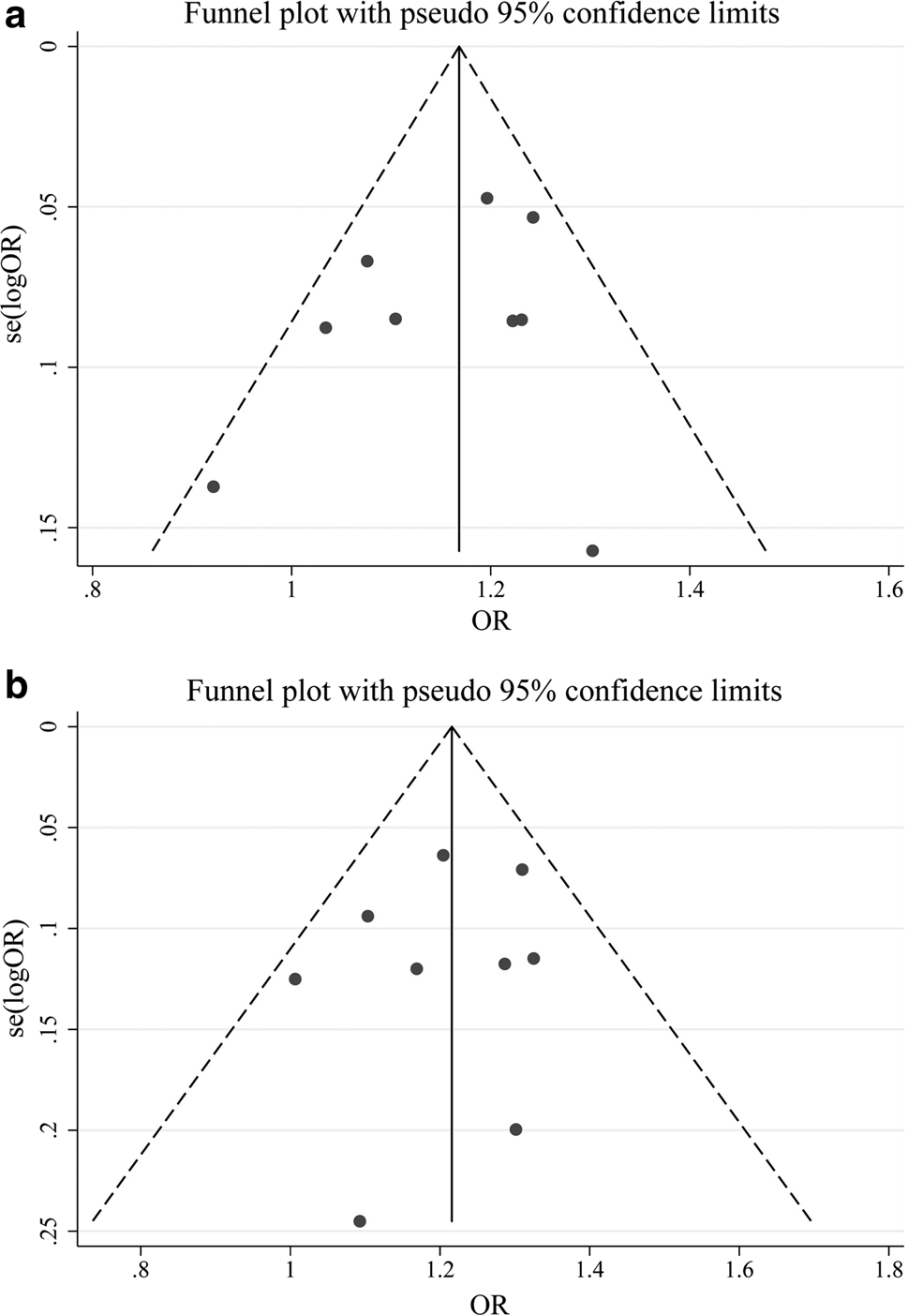
Bias evaluation by funnel plots under **a** an additive model and **b** a recessive model

Potential publication bias in this meta-analysis was examined by funnel plots and Galbraith’s tests. Our results demonstrated that there was no sharp asymmetry in the additive, recessive and dominant models (Table 2, Fig. 2). The results showed no evidence of obvious potential bias among the included publications.

**Fig. 3:**
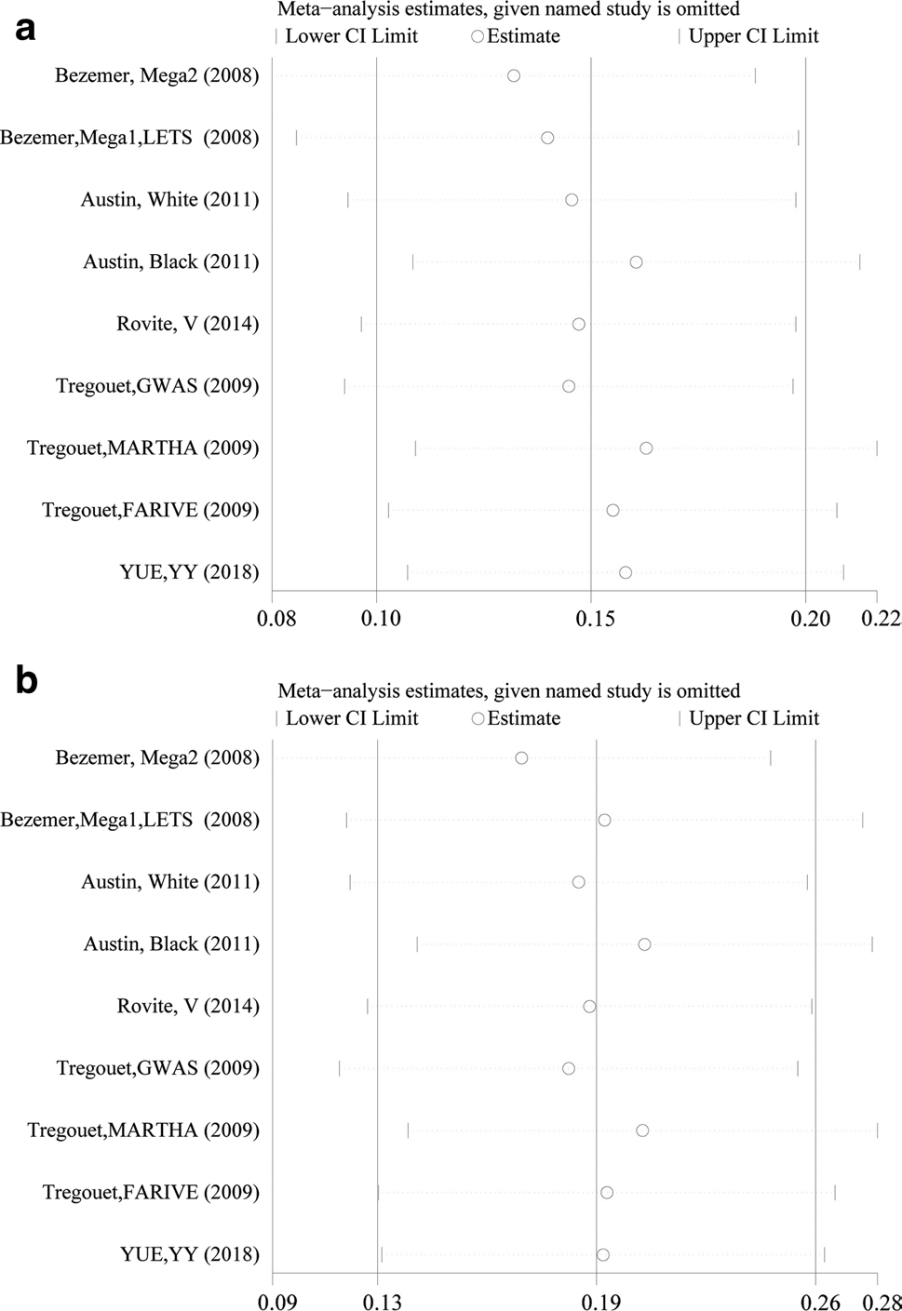
Sensitivity analysis output for the rs13146272 polymorphism under **a** an additive model and **b** a recessive model

Moreover, the sensitivity analyses of all genetic models showed that the pooled OR fluctuated among confidence intervals. Similar results and conclusions were shown, and no results were markedly altered before and after sensitivity analyses (Fig. 3). Collectively, the results suggested that our results and analysis methods were reliable and statistically robust.

## Discussion

In this study, we investigated the association between rs13146272 and VTE risk in the Chinese population. Our case–control study results showed no significant association between VTE and rs13146272. However, when we combined our results with the previous studies for the meta-analysis, the results demonstrated that the association between rs13146272 and VTE risk varied among different ethnicities. Our meta-analysis demonstrated that the *CYP4V2* polymorphism (rs13146272) increased the risk of VTE, and the OR was 1.17 with the additive model (95% CIs: 1.11–1.22, *p* = 4.3e^−10^), was 1.23 with the dominant model (95% CIs: 1.12–1.36, *p* = 2.6e^−5^) and was 1.21 with the recessive model (95% CIs: 1.13–1.29, *p* = 1.1e^−8^). Our results showed that the additive and recessive models reached the threshold for genome-wide significance (*p* < 5.0e^−08^).

As VTE is a complex disease that involves various risk factors, several SNPs, such as factor V Leiden (F5 rs6025), prothrombin 20210G > A (F2 rs1799963) and *CYP4V2* (rs13146272), have been found to increase the risk of developing VTE [31, 32]. *CYP4V2*, located on chromosome region 4q35 and on genes that are involved in coagulation, was reported to be associated with corneal dystrophy disease and lipid metabolism [18, 33]. The association between *CYP4V2* variants and DVT was initially reported in a GWAS study [19]. Another study also showed that 5 SNPs, including rs13146272 from the *CYP4V2* region, were also associated with factor XI (*F11*) levels in some groups [19]. Carriers of risk-increasing alleles rs13146272 showed higher factor XI levels, which implied that the association between DVT and rs13146272 in *CYP4V2* may be coincident with *F11* SNPs. Our study showed that the polymorphisms of rs13146272 only conferred a modest increase in the risk of developing VTE, which indicated that the genetic factors contributing to VTE development may be caused by multiple variants.

The findings regarding the association between rs13146272 and VTE were inconsistent among different studies, especially among different ethnicities. A genome-wide association study only partly confirmed the association of the *CYP4V2* polymorphism rs13146272 with VTE, which was consistent with a replication study in white Americans [20, 21]. The previous meta-analysis showed a significant association between rs13146272 and VTE [20], which was consistent with our results. However, that meta-analysis only included 5 studies, and the analysis was only conducted with the additive model. The other meta-analysis study showed a non-significant conclusion for the pooled overall OR when removing the groups from Bezemer et al.’s study [24]. In our study, the meta-analysis was performed by using three different genetic models and showed a significant association between rs13146272 and VTE. The major A allele of the variant increased the risk of developing VTE, and this finding was consistent among these models. Collectively, the results of our study confirmed a significant association between rs13146272 and VTE based on the meta-analysis with a large sample size.

Our study has several limitations. First, the included studies were based on different populations. The sources of subjects were different, and the methods for analysing aggregated data were difficult to extrapolate. Austin et al. [20] showed that the prevalence of *CYP4V2* risk alleles among white and black controls was different, but the difference was not statistically significant. In fact, different ethnicities carried genetic diversities and had specific characterizations. Second, subgroup analysis of the association between *CYP4V2* and VTE was not performed due to the limited studies for some ethnicities. Third, the genotype detection methods for *CYP4V2* differed among the included studies. Thus, these factors could partially contribute to the heterogeneity among the studies. We used very strict recruitment criteria for young healthy individuals. Each subject did not have any cardiovascular diseases among three generations of siblings and were healthy according to physical examination. All of the subjects had a very low possibility of carrying genetic risk factors for VTE. Even though the mean age was significantly different between the VTE and control groups, the effect of age on genetic diversity was very trivial.

In conclusion, our pooled systematic study results indicated that individuals with the A allele had a higher risk of developing VTE than those with the C allele of rs13146272 variant, but this difference was inconsistent among various ethnicities. *CYP4V2* likely plays an important role in developing VTE. The actual role of VTE-associated variants should be investigated in larger populations with multi-ethnic subjects in the future.
